# Neutrophil-to-Lymphocyte and Platelet-to-Lymphocyte Ratios Are Correlated with Complicated Diverticulitis and Hinchey Classification: A Simple Tool to Assess Disease Severity in the Emergency Department

**DOI:** 10.1155/2019/6321060

**Published:** 2019-08-14

**Authors:** Amir Mari, Tawfik Khoury, Ahmad Lubany, Mohammad Safadi, Moaad Farraj, Amir Farah, Anas Kadah, Wisam Sbeit, Mahmud Mahamid

**Affiliations:** ^1^Gastroenterology and Endoscopy United, The Nazareth Hospital, EMMS, Nazareth, Israel, Bar-Ilan University, Ramat Gan, Israel; ^2^Faculty of Medicine in the Galilee, Bar-Ilan University, Safed, Israel; ^3^Department of Gastroenterology, Galilee Medical Center, Nahariya, Israel; ^4^Department of Surgery, The Nazareth Hospital, EMMS, Nazareth, Israel; ^5^Department of Surgery, Galilee Medical Center, Nahariya, Israel

## Abstract

**Background and Aim:**

Rapid identification of patients with complications related to acute diverticulitis who require urgent intervention in the emergency department (ED) is essential. The aim of our study was to determine the role of neutrophil-to-lymphocyte ratio (NLR) and platelet-to-lymphocyte ratio (PLR) in predicting severity of diverticulitis as assessed by Hinchey classification.

**Patients and Methods:**

We performed a single retrospective study in EMMS Nazareth Hospital from 4/2014 to 4/2018. Patients were categorized into two groups: group A with mild to moderate complicated diverticulitis (Hinchey 1-2) and group B with severe complicated diverticulitis (Hinchey 3-4).

**Results:**

Two hundred twenty-five patients were included. Two hundred seven patients were in group A, and 18 patients were in group B. On univariate analysis, age, NLR, and PLR correlated with advanced Hinchey classification and disease severity (stages 3-4) (OR 1.038, 95% CI 1.001–1.076, *P*=0.0416; OR 1.192, 95% CI 1.093–1.300, *P* < 0.0001; and OR 1.011, 95% CI 1.005–1.017, *P*=0.0005, respectively). On multivariate logistic regression analysis, the NLR and PLR remain significantly correlated with Hinchey 3-4 (OR 1.174, 95% CI 1.071–1.286, *P*=0.0006, and OR 1.008, 95% CI 1.001–1.015, *P*=0.0209, respectively). The area under the curve (AUC) for the NLR and PLR on univariate analysis was 0.7526 and 0.6748, respectively, and 0.7760 and 0.7391 on multivariate logistic regression analysis, respectively, and receiver-operating characteristic (ROC) curves were drawn.

**Conclusion:**

The NLR and PLR independently associated with diverticulitis severity and positively correlated with advanced Hinchey classification. This simple available laboratory tool can be implemented into clinical practice to optimize patient management.

## 1. Introduction

Diverticulosis is an increasingly encountered condition in the western countries. Its prevalence raises with age for about 60% of patients at the age of 80 years [1, 2]. Diverticulosis can be associated with several complications, including acute uncomplicated diverticulitis, painless bleeding, perforation, and segmental colitis associated with diverticulitis. Acute uncomplicated diverticulitis which is an inflammation of the colonic mucosa is the most common complication, as it occurs in 1–25% of patients [3, 4]. Colonic perforation due to diverticular disease has been historically assessed by Hinchey classification as proposed by Hinchey and colleagues [5]. Hinchey classifies perforations to grades: Hinchey I, localized abscess (paracolonic); Hinchey II, pelvic abscess; Hinchey III, purulent peritonitis (the presence of pus in the abdominal cavity); and Hinchey IV, feculent peritonitis. Complicated diverticulitis incidence is believed to increase significantly in the United States since the 1990s [6]. Rapid identification of patients with acute diverticulitis who require urgent intervention in the emergency department (ED) is essential for decision-making [7]. Computed tomography (CT) plays an important role in confirming diverticulitis, evaluating complications, and excluding alternative diagnoses in the ED [8].

Inflammatory markers, such as C-reactive protein (CRP) levels and white blood cell (WBC) count, were shown previously to predict surgical needs in patients with acute diverticulitis [9, 10]. The neutrophil-to-lymphocyte ratio (NLR) is obtained by dividing the absolute number of neutrophils by the absolute number of lymphocytes, and the platelet-to-lymphocyte ratio (PLR) has been used as a prognostic factor in infectious, inflammatory, and malignant diseases [11]. The neutrophils and lymphocytes are white blood cell subsets that play an important role in regulating the inflammatory response in several disease states. The NLR has been shown to be a good prognostic factor in infectious diseases [12]. Furthermore, the NLR and PLR were shown to be better markers of bacteremia severity assessment compared to CRP levels, WBC count, or absolute neutrophil count [13]. The NLR was also shown to be a significant parameter in several disease states including community-acquired pneumonia, myocardial infarction, appendicitis, and several malignancies [14–16]. Furthermore, its use as a prognostic marker to predict complications following colorectal and major abdominal surgeries has been recently described in the literature [17, 18].

The primary aim of this study is to assess the potential role of the NLR and PLR in predicting severity of acute diverticulitis in the ED setting, as assessed by Hinchey classification.

## 2. Patients and Methods

We performed a retrospective cohort study based on files of patients who are admitted to the EMMS hospital with an episode of acute diverticulitis, in the time period of 4/2014 to 4/2018. The patients were identified according to the ICD-9 code and were extracted from the central EMMS hospital archive section. Inclusion criteria included patients older than 16 years of age and confirmed diagnosis of acute diverticulitis as assessed by computed tomography scan. Exclusion criteria included a history of inflammatory bowel conditions, such as inflammatory bowel disease, collagenous colitis, microscopic colitis, and eosinophilic colitis, patients with oncological diseases, and patients with immunosuppressive therapy.

Electronic patients' files were searched for demographics (age, gender, and history of constipation and laxative use), clinical parameters (temperature and location of diverticulitis), and laboratory parameters (WBC, hemoglobin (Hb), platelets, serum urea, and creatinine) (see Table 1). Moreover, we assessed the diverticulitis severity score (Hinchey classification) as assessed by CT scan, and correlation of this score with disease severity and the need for surgical intervention was evaluated. Patients who presented with class I (confined pericolic abscess) and class II (pelvic abscess) were considered as having mild to moderate complicated acute diverticulitis and referred to group A. Patients with class III (generalized purulent peritonitis) and class IV (generalized fecal peritonitis) were considered to have severe complicated diverticulitis and referred to group B. Comparisons between the two groups were performed. This study was approved by the local institutional ethics committee. Written informed consent was waived because of the retrospective noninterventional design of this study.

## 3. Study Endpoints

Primary endpoints were to evaluate the association of the NLR and PLR with severity of acute diverticulitis as assessed by Hinchey classification and to examine whether the NLR and PLR were correlated with the increased rate of surgical intervention.

## 4. Statistical Analysis

Chi-square and Fisher's exact tests were used to analyze the association between two categorical variables which were presented as frequencies and percentages, while either the two-sample *t*-test or the Mann–Whitney *U* test was used to compare continuous variables. All *P* values were two-sided, and statistical significance was set at *P* < 0.05. Variables with a statistically significant value with *P* values <0.05 by univariate analysis were entered into the multivariate logistic regression analysis. The NLR and PLR were tested through area under the curve (AUC) by receiver-operating characteristic (ROC) curve analysis to determine the association of the NLR and PLR with advanced Hinchey classification. The AUC was reported in univariate and multivariate analysis. In addition, we determined the optimal cutoff points for both ratios using ROC curves and the Youden index. Moreover, we generated several cutoff points for both ratios and their correlated sensitivity and specificity for correlation with advanced Hinchey classification. Statistical analysis was performed using SPSS Version 23 (IBM, Armonk, NY, USA).

## 5. Results

### 5.1. Demographics and Clinical and Laboratory Characteristics

Two hundred twenty-five patients with CT-confirmed acute diverticulitis were included. Table 1 summarizes the patients' baseline characteristics. Two hundred seven patients were in group A (Hinchey 1-2), and 18 patients were in group B (Hinchey 3-4). The mean age in group A was 55.17 ± 13.38 years as compared to 62.17 ± 17.26 years in group B. One hundred twenty-seven patients (56.4%) in group A were male as compared to 12 patients (66.7%) in group B. There was no difference in the location of diverticulitis. Similarly, there was no difference in the basic laboratory test (WBC, Hb, platelets, and kidney function) between the two groups.

### 5.2. Parameters Associated with Advanced Hinchey Classification on Univariate and Multivariate Logistic Regression Analysis

In a univariate model analysis, we found that age, NLR, and PLR correlated with advanced Hinchey classification (*P*=0.0416, <0.0001, and 0.0005, respectively), while only a trend for correlation was seen with the serum creatinine level (*P*=0.0808), urea level (*P*=0.0887), and constipation (*P*=0.0984) (Table 2). In multivariate logistic regression analysis, the NLR and PLR showed significant correlation (odds ratio (OR) 1.174, 95% CI 1.071–1.286, *P*=0.0006, and OR 1.008, 95% CI 1.001–1.015, *P*=0.0209, respectively).

### 5.3. Receiver-Operating Characteristic (ROC) Curve Analysis of NLR and PLR

**Figure 1:**
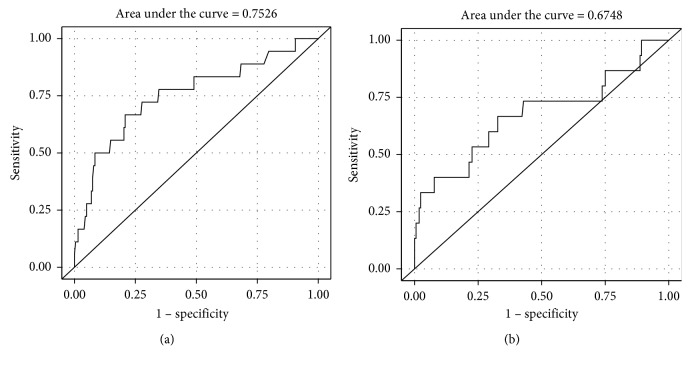
ROC curve with AUC for the NLR (a) and PLR (b) on univariate analysis for severity of diverticulitis.

**Figure 2:**
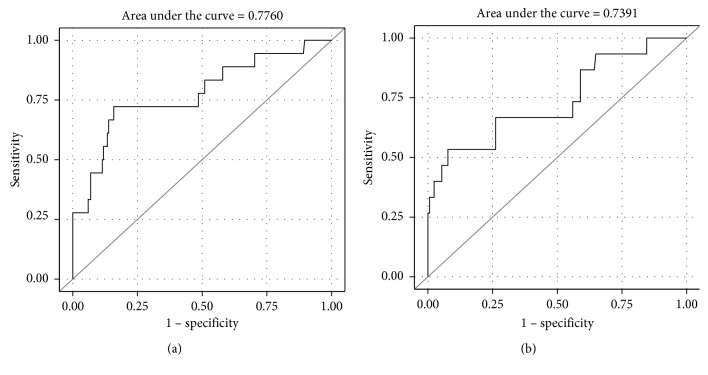
ROC curve with AUC for the NLR (a) and PLR (b) on multivariate regression analysis for severity of diverticulitis.

We performed ROC curve analysis for the NLR and PLR which we used to define a threshold above which the NLR and PLR may predict diverticulitis severity. The threshold was defined for the value with maximal sensitivity and specificity. The AUC for the NLR and PLR on univariate analysis was 0.7526 and 0.6748, respectively (Figure 1). On multivariate logistic regression analysis, the AUC for the NLR and PLR was 0.7760 and 0.7391, respectively (Figure 2), and ROC curves were drawn for the NLR and PLR. When examining several cutoff points for the NLR that showed correlation with advanced Hinchey classification, we found that the Youden index (*J*) was >6.68 which has a sensitivity of 68.75% and a specificity of 79.21%. Other NLR cutoff points with their corresponding sensitivity and specificity are shown in Table 3. Similarly, the Youden index (*J*) of the PLR was >139.15 that has a sensitivity and specificity of 76.92% and 67.26, respectively. Other PLR cutoff points with their corresponding sensitivity and specificity are shown in Table 4. Moreover, we found that the NLR and PLR showed significant positive correlation with the need for surgical intervention in patients with acute diverticulitis, and the averages of the NLR and PLR in patients who needed surgery during the hospitalization were 8.23 and 200.35 as compared to 5.18 and 125.79 in patients who were treated conservatively (*P*=0.016 and 0.0009, respectively).

## 6. Discussion

The primary aim of our study was to assess the association of the NLR and PLR with Hinchey classification which is used to evaluate the severity of acute diverticulitis with its related complications. On univariate analysis, the NLR and PLR showed a statistically significant association with advanced Hinchey classification. Moreover, on multivariate regression analysis, this association was kept and was shown to be independent from the other variables. The neutrophils and lymphocytes are important components of the white blood cells which play an important role in the regulation of the inflammatory microenvironment and can be reflected in peripheral blood measurable parameters. The NLR was reported in a previous study as a reliable marker for differentiating patients presenting to the emergency room with sepsis [12]. Furthermore, the NLR was shown to be a reliable marker for bacteremia severity assessment among adult hospitalized patients [13]. The NLR was also shown to be a significant parameter in several disease conditions including community-acquired pneumonia, acute myocardial infarction, complicated appendicitis, and colorectal cancer [14–17]. Furthermore, its use as a prognostic marker to predict complications following a variety of major surgical procedures has been recently described in the literature [18, 19]. Recently, the NLR has been shown to have a role in the prognosis of patients with cirrhosis [20].

Only one recent study has reported the association of the NLR with surgical intervention in patients with acute diverticulitis [10]. Similarly, in our study, we showed that the NLR and PLR were independently associated with increased surgical intervention. This positive correlation is reflected by the correlation of the NLR and PLR with advanced Hinchey classification which indicates the presence of severely complicated acute diverticulitis that necessitates surgery. The rationale behind this high NLR might be explained in part by the development of neutrophilia and relative lymphopenia as a result of diffuse systemic septicemia and bacterial infections [21]. Thus, it was suggested that the ratio between neutrophils and lymphocytes is more precise in predicting poor clinical and surgical outcomes than either parameter alone [18]. Our study is the first to show the positive association of the NLR with advanced Hinchey classification and subsequently predicts the need for surgical intervention. Platelets are an important factor in mediating coagulation, thrombosis, and inflammation as they secrete several molecules involved in inflammation [22]. Moreover, the platelet's role in identifying the inflammatory processes is characterized by regulation of other types of cells such as neutrophils and facilitation of their adhesion to lymphocytes and also by promotion of the growth and spread of malignancies via oncoinflammatory mechanisms [23, 24]. To date, the PLR has been shown as an important prognostic factor in several malignancies [25–29]. However, no studies have reported the prognostic role of the PLR in acute diverticulitis; we found that the PLR as well is correlated with the advanced Hinchey score and need for surgery. Moreover, we generated several ROC cutoff points with their corresponding sensitivity and specificity that might be a useful addition to improve patients' evaluation and assess severity of complicated diverticulitis in patients presenting to the ED.

The mean age in the advanced Hinchey classification group was significantly higher than that in the mild to moderate Hinchey group (*P*=0.0416) on univariate analysis; however, the significance was lost on multivariate regression analysis (OR 1.015, 95% CI 0.975–1.057, *P*=0.45), and still we believe that older age might be a risk factor for complicated diverticulitis because of the atypical diverticulitis-related symptoms coupled with the attenuated physiological defense mechanisms of the gastrointestinal tract. The main limitation of our study is its retrospective nature of data collection and that it was conducted in a single center. On the contrary, the strengths include the relatively larger cohort number and the presence of advanced imaging tools (CT scan) that accurately evaluated the complications of diverticulitis.

In conclusion, our study demonstrated that the NLR and PLR have significant correlation with severe complicated acute diverticulitis as assessed by Hinchey classification. We suggest implementing our findings into clinical assessment and decision-making regarding emergent treatment and surgical intervention among patients with acute diverticulitis presenting to the emergency room. Further prospective trials are warranted to be performed to confirm our preliminary findings.

## Figures and Tables

**Table 1:** Demographics and characteristics of the study cohort.

Parameters	Group A (Hinchey 1-2)	Group B (Hinchey 3-4)
Number of patients	207	18
Age (years) (mean ± SD (range))	55.17 ± 13.38 (24–93)	62.17 ± 17.26 (24–93)
Gender, *N* (%)		
Male	127 (61.3)	12 (66.7)
Female	79 (38.2)	6 (33.3)
Location of diverticulitis, *N* (%)		
Right colon	14 (6.7)	2 (11.1)
Left colon	187 (90.3)	15 (83.3)
History of abdominal surgery, *N* (%)	42 (20.3)	5 (27.8)
Preadmission constipation	14 (6.7)	3 (16.6)
Preadmission laxative use	7 (3.4)	0
Preadmission aspirin use	40 (19.3)	4 (22.2)
Temperature (°C) (mean ± SD (range))	36.7 ± 0.53 (36–39)	36.7 ± 0.46 (36–37.8)
White blood cell count (mean ± SD)	11684.36 ± 4606.89	13555.29 ± 6357.25
Hemoglobin (mean ± SD)	13.90 ± 1.94	13.21 ± 2.32
Creatinine (mean ± SD)	0.99 ± 1.10	1.62 ± 2.01
Urea (mean ± SD)	14.67 ± 12.03	21.00 ± 20.93
Platelets (mean ± SD)	241436.89 ± 70082.14	256111.11 ± 73394.09

**Table 2:** Univariate analysis of parameters positively or negatively associated with abscess formation.

Parameters	Odds ratio	95% CI for odds ratio	*P* value
Age	1.038	1.001–1.076	0.0416
NLR	1.192	1.093–1.300	<0.0001
PLR	1.011	1.005–1.017	0.0005
Creatinine	1.242	0.974–1.584	0.0808
Urea	1.021	0.997–1.045	0.0887
Constipation	3.014	0.815–11.150	0.0984

**Table 3:** ROC analysis of NLR cutoff values and their association with the advanced Hinchey score.

NLR cutoff value	Sensitivity (%)	Specificity (%)
0.78–1.4	100	0–9.4
1.5–5	81–94	10–65
>5–8	62–75	66–85
>8–10.5	50–56	86–92
>10.5–13.5	20–44	92–96
>21.5	6.2	100

**Table 4:** ROC analysis of PLR cutoff values and their association with the advanced Hinchey score.

PLR cutoff value	Sensitivity (%)	Specificity (%)
3–59	100	0–10.7
60–124	84.5–92.3	10.7–57
125–153	61.5–77	57.7–77.4
154–266	30.7–53.8	77.38–98.21
>361	0–23	100

## Data Availability

The data used to support the findings of this study are available from the corresponding author upon request.

## Abbreviations

ED: Emergency department
CT: Computed tomography
CRP: C-reactive protein
WBC: White blood cell
NLR: Neutrophil-to-lymphocyte ratio
PLR: Platelet-to-lymphocyte ratio
Hb: Hemoglobin
AUC: Area under the curve
ROC: Receiver-operating characteristic
OR: Odds ratio
CI: Confidence interval.
